# Guy's Hospital: A Case of Rupture of the Bladder

**Published:** 1897-09-04

**Authors:** L. A. Dunn

**Affiliations:** Assistant-Surgeon to the Hospital


### Medical Progress and Hospital Clinics.

*[The Editor will be glad to receive offers of co-operation and contributions from members of the profession. All letters should be addressed to* The Editor, at the Office, 28 & 29, Southampton Street, Strand, London, W.C.]

### Notes of Hospital Clinics.

*[Reported for* The Hospital, *and corrected by the authors.]*

### GUY'S HOSPITAL.

A Case of Rupture of the Bladder.

By L. A. Dunn, M.S., F.R.C.S., Assistant-Surgeon to the Hospital.

The case we have to consider to-day is that of a bricklayer's labourer, aged 34, who was brought up to the surgery at Guy's Hospital, late in the afternoon. It appeared that he had been indulging in alcoholic stimulants rather too freely, and that whilst walking upon a wall some twelve feet high he had overbalanced and fallen to the ground prone. When he was seen by the house surgeon he was excited with alcohol, and complained of nothing except that he wanted to pass water, and was unable to do so. A catheter was passed into the bladder, and about two ounces of bright blood were withdrawn. The dresser who passed the catheter noticed that it entered an undue distance. Rupture of the urinary bladder was diagnosed, and the patient was straightway admitted into the accident ward. It was then found that both knees were contused, and that the right carpus was dislocated forward from the radius. There were also some superficial abrasions of the nose and forehead. The dislocation was easily reduced, and I was called in to see the case in the absence of Mr. Golding Bird. The patient was still rather alcoholic, although he had become somewhat sobered at the sight of the blood which had been drawn off. The pulse was under 100 per minute, and of good power, and the patient expressed himself as feeling quite right except for the desire to pass water. I passed a catheter with a stilette easily into the bladder, having previously sterilised it and cleaned the meatus with lysol. On withdrawing the stilette about an ounce of deeply blood-stained urine ran out in a good stream, which was not affected by the respiratory movements. Something was felt to flip against the end of the instrument, and the stream instantly ceased. On rotating the catheter the stream of urine again flowed with the same force, evacuating a similar amount. On withdrawing the catheter the eye was found to be quite clear. Previously to passing the catheter the abdomen was examined, and was quite supple and perfectly resonant over the hypogastrium. There was no tenderness. On rectal palpation nothing abnormal could be discovered, but the prostate was thought to be rather too large for a man of the patient's age. After the urine had been drawn off, the desire to micturate ceased to a great extent. I could not make the catheter pass to any undue length into the bladder.

As it was thought that the symptoms could be accounted for by the fact that the bladder had been filled with blood which was now being broken up by the urine, it was decided to wait till the next day, and have the bladder relieved at intervals by means of the catheter. The dresser did this during the night, drawing off from ʒi to ʒiijs on each occasion.

On the first occasion the urine came away fairly clear, but he experienced the same sudden stoppage that I had done, and on withdrawing the instrument he found the eye blocked with blood clot.

On Wednesday, at Mr. Golding Bird's request, I saw the patient again late in the afternoon. The abdomen was distended and tympanitic all over, and it was tender on pressure, especially over the hypogastrium. The pulse was under 100, and of good quality. He was sick at times, bringing up a little of the stomach contents, but he took plentifully of milk. The urine had been drawn off during the day, but amounted to only a small quantity. I thought so seriously of the case that I had the abdomen cleaned and shaved, and covered with a compress of perchloride of mercury. A catheter was passed and tied in the bladder.

The next morning—Thursday—the patient was feeling much better, which he attributed to the application of the compress. The temperature, which had ranged from 99 deg. to 100 deg., was now normal. The pulse was 88. He had lost all pain, and the abdomen was quite supple, and no longer distended. He had passed flatus freely during the night, and about a pint of clear urine had drained away through the catheter. The vomiting had ceased, and he was taking farinaceous diet. I saw the case with Mr Golding Bird in the afternoon, and we were both agreed that all operation ought to be postponed. The improvement was maintained until Saturday, when he was again slightly sick, and the abdomen became again distended. Towards evening an enema of castor oil and turpentine was administered without marked result.

On Sunday morning at nine a.m., when I saw him, his condition had markedly changed. His face had a bluish tinge, his extremities were cold, the pulse was over 120 and very feeble, and the abdomen was greatly distended, resonant all over (indeed, there never had been any dulness, either over the hypogastrium or elsewhere), and tender on pressure over the hypogastrium. His condition was so grave that I considered a laparotomy would prove immediately fatal, so stimulants by the mouth, hot blankets, and stimulating nutrient enemata were ordered in the hope that he might rally sufficiently by the evening to bear the operation. The compress was again applied.

In the evening he was somewhat stronger, and as it seemed clear that he would not survive the night if nothing was done, the operation was decided on. I had no doubt now that there was a collection of urine in the peritoneal cavity by which he was being poisoned, and my object in operating was to open and drain this collection as quickly as possible without making any attempt to suture the rent in the bladder, as it was quite clear that the operation must be as rapid as possible.

An attempt was made to wash out the stomach, as the stomach was greatly distended, and it was thought that if vomiting occurred under the anæsthetic it would certainly prove fatal. This attempt, however, failed. The A.C.E. mixture was administered, and an incision about four inches long, down to and through the peritoneum, was made in the median line, ending at the symphysis pubis. The subjacent intestines were found glued together with recent lymph. They were quickly separated with the finger tips in the direction of the bladder, and a large collection of foul urine was evacuated. The cavity was washed out with hot water poured from a jug. The bladder was felt with the exploring finger, but no rent was detected. Broad strips of iodoform gauze were placed in the wound, reaching to the bottom of the pelvis. The upper part of the wound was closed with two sutures, and dressings applied. The whole operation lasted only a few minutes, thanks to the able assistance of the house surgeon and dressers. The patient, however, never rallied, and died shortly after the operation.

At the post-mortem examination there was found no general peritonitis. The cavity behind the bladder—the walls of which were formed by agglutinated intestines and the bladder—had been completely drained. The interior of the bladder was bloodstained, and a rent was found about an inch long in the mucous membrane at the upper and left part posteriorly; this led into a small space in the cellular tissue, which opened into the recto-vesical pouch of peritoneum. The rents in the bladder and peritoneum consequently did not exactly correspond.

This was a very difficult case for diagnosis. The entire absence of shock, and the fact that the urine flowed through the catheter in a forcible stream not influenced by respiratory movements, made me think that ruptured bladder was unlikely; in the same way the absence of rigidity of the abdomen and dulness over the hypogastrium were misleading. It seemed possible hat some rupture of the kidney or laceration of the mucous membrane might account for the blood in the bladder. I imagine the valve-like arrangement of the rent in the bladder-wall accounts for the force of the stream of urine. The post-mortem appearance bore out the observations of Professor Coates that healthy urine does not produce an acute general peritonitis. Had I operated earlier, I have little doubt but that the patient's life would have been saved. Then, on the other hand, the symptoms were so vague and uncertain that I think the delay was to a great extent justified.

